# Genome-Wide Characterization and Expression Analysis of Soybean TGA Transcription Factors Identified a Novel TGA Gene Involved in Drought and Salt Tolerance

**DOI:** 10.3389/fpls.2019.00549

**Published:** 2019-05-16

**Authors:** Bo Li, Ying Liu, Xi-Yan Cui, Jin-Dong Fu, Yong-Bin Zhou, Wei-Jun Zheng, Jin-Hao Lan, Long-Guo Jin, Ming Chen, You-Zhi Ma, Zhao-Shi Xu, Dong-Hong Min

**Affiliations:** ^1^College of Agronomy, Northwest A&F University/State Key Laboratory of Crop Stress Biology for Arid Areas, Yangling, China; ^2^Institute of Crop Science, Chinese Academy of Agricultural Sciences (CAAS)/National Key Facility for Crop Gene Resources and Genetic Improvement, Key Laboratory of Biology and Genetic Improvement of Triticeae Crops, Ministry of Agriculture, Beijing, China; ^3^College of Life Sciences, Jilin Agricultural University, Changchun, China; ^4^College of Agronomy, Qingdao Agricultural University, Qingdao, China

**Keywords:** soybean, TGA transcription factor, molecular characterization, abiotic stress response, drought and salt tolerance

## Abstract

The TGA transcription factors, a subfamily of bZIP group D, play crucial roles in various biological processes, including the regulation of growth and development as well as responses to pathogens and abiotic stress. In this study, 27 *TGA* genes were identified in the soybean genome. The expression patterns of *GmTGA* genes showed that several *GmTGA* genes are differentially expressed under drought and salt stress conditions. Among them, *GmTGA17* was strongly induced by both stress, which were verificated by the promoter-GUS fusion assay. *GmTGA17* encodes a nuclear-localized protein with transcriptional activation activity. Heterologous and homologous overexpression of *GmTGA17* enhanced tolerance to drought and salt stress in both transgeinc *Arabidopsis* plants and soybean hairy roots. However, RNAi hairy roots silenced for *GmTGA17* exhibited an increased sensitivity to drought and salt stress. In response to drought or salt stress, transgenic *Arabidopsis* plants had an increased chlorophyll and proline contents, a higher ABA content, a decreased MDA content, a reduced water loss rate, and an altered expression of ABA- responsive marker genes compared with WT plants. In addition, transgenic *Arabidopsis* plants were more sensitive to ABA in stomatal closure. Similarly, measurement of physiological parameters showed an increase in chlorophyll and proline contents, with a decrease in MDA content in soybean seedlings with overexpression hairy roots after drought and salt stress treatments. The opposite results for each measurement were observed in RNAi lines. This study provides new insights for functional analysis of soybean TGA transcription factors in abiotic stress.

## Introduction

Abiotic stress, such as drought and high salinity, greatly affect plant growth and development. In order to adapt to abiotic stress, plants have evolved complex signal transduction pathways and diverse response mechanisms to protect themselves against cellular damage caused by abiotic stress (Munns and Tester, 2008; Chen and Murata, 2011; Krasensky and Jonak, 2012; Mickelbart et al., 2015; Zhu, 2016; Qi et al., 2018; Yang and Guo, 2018). Under stress conditions, changes in gene expression are the earliest responses in plants, and a number of stress-responsive genes have been noted to have important functions in drought and salt resistance. Among these genes, transcription factors are very important as the proteins they typically encode control the transcription of downstream genes (Singh et al., 2002; Li et al., 2008). Among them, the basic leucine zipper (bZIP) gene family is one of the largest transcription factor families in plants. bZIP genes were classified into 10 groups (A, B, C, D, E, F, G, H, I, and S), along with another two extra groups, J and K, based on the similarity in the basic region and additional conserved motifs (Jakoby et al., 2002; Nijhawan et al., 2008). The TGA (TGACG motif-binding factor) transcription factors belong to group D, which can recognizes *as-1-* type *cis*-elements located in the promoter region of the target genes (Katagiri et al., 1989; Zhang et al., 1999; Johnson et al., 2003). Tobacco TGA1a was the first TGA transcription factor cloned from plants and is characterized by the conserved basic region/leucine zipper domain (Katagiri et al., 1989). Subsequently, more TGA transcription factors were identified in various plants (Jakoby et al., 2002; Espín et al., 2012). For TGA proteins, the primary structure of the bZIP domain is conserved, containing an invariant motif N-x_7_-R/K-x_9_-L-x_6_-L-x_6_-L in the N-terminus, and a bZIP-D box, the motif Yx_2_RL[RQ]ALSS[LS]W, represents the signature domain of group D in the C-terminus (Jakoby et al., 2002).

A growing body of evidence has shown that members of TGA transcription factor are known to play crucial roles in many biological processes: defense against pathogens and plant development (Johnson et al., 2001; Thurow et al., 2005; Choi et al., 2010; Zander et al., 2012; Gatz, 2013; Wang et al., 2016). Despite this body of work, little has been reported about the functions of TGA transcription factors in plant responses to abiotic stress. The *Brassica juncea BjCdR15*, an orthologous gene of *Arabidopsis TGA3*, had an induced expression in response to cadmium and stress from other heavy metals (Fusco et al., 2005). Transgenic *Arabidopsis* and tobacco overexpressing *BjCdR15* exhibited an enhanced tolerance to cadmium through regulation of cadmium uptake and long-distance transport (Farinati et al., 2010). The crab apple *MhTGA2*, which showed an induced expression in response to low temperature, NaCl, and PEG, enhanced tolerance to salt and osmotic stress in transgenic apple and tobacco (Zhang et al., 2012; Du et al., 2014). Overexpression of *AtTGA4* increased tolerance to drought stress by improving nitrate transport and absorption in *Arabidopsis* (Zhong et al., 2015). These findings suggested that TGA transcription factors may play functions in plant adaptation to various abiotic stimuli, including drought and salt.

Soybean (*Glycine max* L.), an economically important oil and protein crop, is considered a moderately drought- and salt-tolerant plant, though its growth and productivity is adversely affected by soil drought and salinity. Given the potential importance of *TGA* genes in plant responses to abiotic stress, we conducted a genome-wide analysis of the soybean TGA family and investigated the potential functions of *TGA* genes in plant responses to drought and salt stress.

## Materials and Methods

### *In silico* Identification of Soybean TGA Transcription Factors

The sequences of known TGA proteins from *Arabidopsis* were retrieved from the TAIR database. These sequences were used as queries to search against the soybean genome database using the BLASTP program with a threshold *E*-value cutoff of 1.0 × e^-5^ (Espín et al., 2012). Then, each candidate soybean TGA sequence used as a query against the Pfam database to confirm its membership in the bZIP family (Finn et al., 2016). Repeat sequences were removed manually. The ExPASy server was used to predict several physio-chemical parameters of TGA proteins such as molecular weights (Mw) and theoretical isoelectric points (p*I*) (Gasteiger et al., 2003).

### Sequences Analysis

The exon/intron gene boundaries were analyzed using the Gene Structure Display Server 2.0 (GSDS) tool (Hu et al., 2015). The 2.0 kb sequences upstream of the start codon of soybean *TGA* genes were extracted from the phytozome database as the regulatory promoter region. Putative *cis*-acting elements were analyzed using the PlantCARE database (Lescot et al., 2002). Multiple sequence alignments for the predicted protein sequences were performed using ClustalX software (Thompson et al., 1997). MEGA6.0 software was used to construct a phylogenetic tree based on the bootstrap neighbor-joining (NJ) approach followed by 1000 bootstrap replicates (Tamura et al., 2013).

### Expression Analysis of Soybean *TGA* Genes in Different Tissues

The analysis of gene expression patterns in different tissues, including roots, root hairs, stem, leaves, nodules, seeds, and flowers, was carried out using transcriptome data obtained from the Phytozome database, and the heatmap was produced with HemI software (Deng et al., 2014).

### Plant Materials, Growth Conditions and Stress Treatments

The soybean variety Williams 82 was used for experiments in this study. Seedlings were grown in pots containing mixed soil (humus:vermiculite = 1:1) in a greenhouse with a 14-h-light/10-h-dark photoperiod, 28/20°C day/night temperatures, and 60% relative humidity. Sixteen-day-old seedlings were subjected to drought and salinity treatments. For drought stress, seedlings were removed from the soil and left to dry on filter paper. For the high-salinity treatment, the roots of seedlings were immersed in solution containing 200 mM NaCl. For treatments with exogenous ABA, leaves were sprayed with 200 μM ABA solution. Seedlings were sampled for RNA extraction at 0, 1, 3, 6, 12, and 24 h after each respective treatment.

### RNA Isolation and Quantitative Real-Time PCR (qRT-PCR)

Total RNA was isolated from soybean seedlings and hairy roots, or from *Arabidopsis* seedlings using Trizol reagent (TaKaRa, Japan). The first strand cDNA was synthesized using the PrimeScript 1st Strand cDNA Synthesis Kit (TaKaRa, Japan) based on the manufacturer’s instructions. qRT-PCR was performed on ABI prism 7500 Real-Time PCR system (Applied Biosystem, United States) using SYBR Green Real Master Mix (Tiangen, China) with the following PCR cycles: 95°C for 15 min, followed by 40 cycles of amplification (95°C for 10 s, 58°C for 20 s, and 72°C for 32 s). The data of qRT-PCR were determined using the 2^-ΔΔCt^ method according to the cycle threshold (*C*t) values (Livak and Schmittgen, 2001). The soybean *CYP2* (*GmCYP2*) (Glyma.12g024700) and *Arabidopsis actin2* (*AtACT2*) (At3g18780) genes were used for normalization. Student’s *t*-test was used to determine significant differences. A level of 0.05 was used for statistical significance. Primer sequences for qRT-PCR analysis were designed using the software tool Primer Premier 5.0. The primer sequences are listed in Supplementary Table S1. All reactions were conducted with four biological replicates for each sample.

### Subcellular Localization and Transcriptional Activation Analysis

The ORF of *GmTGA17*, lacking the stop codon, was amplified using custom primers and fused to the N-terminal region of GFP. The ORF of *NtTGA2.2* (AAF06696), encoding a nuclear-localized protein, was cloned into the N-terminal region of RFP as the positive control (Thurow et al., 2005). The genes were driven by CaMV35S promoter. The reconstruction plasmid of NtTGA2.2-RFP and GmTGA17-GFP were co-transferred into *Arabidopsis* protoplasts, and NtTGA2.2-RFP and the 35S::GFP vector were co-transformed as the control. The fluorescence signal was detected by confocal microscopy (Leica Microsystem, Heidelberg, Germany) after incubating in darkness at 22°C for 16 h. The primers used are shown in Supplementary Table S1.

To perform the transcriptional activity assay in yeast cells, the ORF of *GmTGA17* was amplified and cloned into the pGBKT7 vector. pGBKT7-AtDREB2A was the positive control based on previously reported data (Sakuma et al., 2006), while the pGBKT7 empty vector was the negative control. The plasmids were transformed into yeast strain AH109 according to the method described previously (Gietz and Schiestl, 2007). The Yeastmaker^TM^ Yeast Transformation System 2 (Clontech, United States) was used for yeast transformation. Transcriptional activity was analyzed using methods established in prior work (Yang et al., 2010).

The transcriptional activity was examined in *Arabidopsis* protoplast system. The reporter was a plasmid containing the firefly (*LUC*) gene fused with 5 × GAL4 binding sites under the control of CaMV35S promoter. The other plasmid with the renilla luciferase (*REN*) gene was used as the internal control (Liu et al., 2018). GAL4-BD and BD-AtDREB2A were used as negative and positive effector. The each effector plasmid was co-transformed with the two reporters. Firefly and Renilla luciferase was quantified at 18 h post-transformation by using the Dual-Luciferase Reporter Assay System following the manufacturer’s instructions (Promega, United States).

### Generation of Transgenic *Arabidopsis* Plants

In order to produce transgenic *Arabidopsis* lines, the ORF of *GmTGA17*, the stop codon, was amplified and cloned into the pCAMBIA1302 vector driven by the CaMV35S promoter. The expression vector pCAMBIA1302-GmTGA17 was transformed into *Agrobacterium tumefaciens* strain GV3101 and transferred into *Arabidopsis* Col-0 plants using the floral dip method (Clough and Bent, 1998). The harvested seeds were screened by hygromycin (40 mg/L) resistance. T_3_ transformed plants were confirmed by qRT-PCR analysis and used for further study based on the expression level of *GmTGA17*.

### *Agrobacterium rhizogenes*-Mediated Transformation of Soybean Hairy Roots

To generate *GmTGA17* promoter-GUS construct, a 1.9-kb fragment upstream from the initiation codon was amplified and then used to replace the CaMV35S promoter in pCAMBIA3301. The ORF of *GmTGA17* was amplified and cloned into pCAMBIA3301 under the control of the CaMV35S promoter to generate the pCAMBIA3301-GmTGA17 overexpression vector. For construction of the RNAi suppression vector, a 542 bp ligated fragment containing the sequence from CDS positions 549 to 750, the first intron sequence and the reverse complement sequence of the sequence from CDS positions 549 to 750 (Supplementary Table S1) was synthesized (Biomed, Beijing, China) and cloned into pCAMBIA3301 to generate pCAMBIA3301 -GmTGA17-RNAi vector. All recombinant vectors were transformed into soybean hairy roots by high-efficiency *Agrobacterium rhizogenes*-mediated transformation as described previously (Kereszt et al., 2007).

### Abiotic Stress Tolerance Assessments of Transgenic *Arabidopsis* and Soybean Hairy Roots

Three transgenic *Arabidopsis* lines with a higher expression of *GmTGA17* were used to evaluate the drought and salt tolerance. For the root growth assay, 5-day-old seedlings were transferred to 1/2 MS medium ( containing 6%, 9% or 12% PEG6000 and 50, 75 or 100 mM NaCl, respectively) for vertical growth under a photoperiod of 16-h-light/8-h-dark at 22°C, 40 μmol m^-2^ s^-1^ light. Seedling roots were scanned with Expression 11000XL scanner and analyzed for total root length with WinRHIZO software after 8 days of treatments. For drought stress in soil, water was withheld from 2-week-old soil-grown seedlings until differences in phenotype were observed, then plants were watered again and recovered for 1 week to count the survival rates. For high-salinity treatment, 2-week-old soil-grown seedlings were soaked in 250 mM NaCl solution for 1 week, and the control group continued to grow under normal conditions. Proline and MDA contents were measured under both stress conditions, and chlorophyll content was measured under high-salinity treatment as described previously (Arnon, 1949; Bates et al., 1973; Cakmak and Horst, 1991).

For water loss measurement, the leaves from 3-week-old plants grown in soil under normal conditions were excised and weighted immediately (initial weight, W0). Subsequently, the detached leaves were maintained in a growth chamber (40% relative humidity) and measured at designated time intervals. The fresh weights measured at each time point were used as Wn. Three replicates were done for each line. The water loss rate was calculated as (W0-Wn)^∗^100/W0.

The transgenic hairy roots were confirmed by PCR and qRT-PCR analysis. For promoter-GUS analysis, the transgenic hairy roots were immersed in 10% PEG6000, 100 mM NaCl, or water. The GUS activity and the transcript level of *GUS* in soybean transgenic hairy roots were detected at 0, 3, 6, 9, 12, and 24 h post-treatments. Histochemical staining of GUS was performed as previously described (Jefferson, 1987). In addition, the expression levels of *GUS* were analyzed by qRT-PCR.

For abiotic stress tolerance assays, the transgenic hairy roots with higher (OE) or lower (RNAi) expression level of *GmTGA17* were selected for further study. 10-day-old soil-grown plants with transgenic hairy roots were soaked in 20% (m/v) PEG6000 or 150 mM NaCl solutions for 10 days. The control group continued to grow under normal conditions. After treatments, the transgenic roots were scanned and the total root length and the total root surface were analyzed. Meanwhile, chlorophyll, proline, and MDA contents in leaves were detected as described above.

### Stomatal Aperture Measurement

Stomatal aperture assay was performed as described previously (Kim and Kim, 2013). The guard cells were photographed using confocal microscopy (Leica Microsystem, Heidelberg, Germany), and the stomatal aperture (ration of width to length) was analyzed using Photoshop CS5 software (Adobe System).

### Determination of ABA Content

ABA content was measured as described previously (Liu et al., 2014). The Phytodetek-ABA ELISA Kit (Agdia, United States) was used for the determination of ABA according to the manufacture’s protocol.

### Statistical Analysis

All experiments above were repeated at least three replicates independently. The data were subjected to Student’s *t*-test analysis using functions in Excel 2007. The values are shown as mean ± standard deviation (SD). *P*-value cut-off of 0.05.

### Primers

The primers and sequences used in this study are listed in Supplementary Table S1.

## Results

### Identification of TGA Transcription Factors in Soybean

In this study, full-length proteins and conserved domains of 10 *Arabidopsis* TGAs were used as BLAST query sequences against the soybean genome database. A total of 27 non-redundant, putative *TGA* genes were identified in the soybean genome, which named *GmTGA01-GmTGA27* according to the Gene ID number (Table 1). These loci were distributed across 16 chromosomes in the soybean genome, with the exceptions of chromosomes 7, 9, 16, and 17. The size range of the predicted products of these putative GmTGAs spans 290 (GmTGA05) to 517 (GmTGA17) amino acids residues, with molecular weights (Mw) ranging from 32.37 kDa (GmTGA05) to 57.92 kDa (GmTGA11), and protein isoelectric points (p*I*s) ranging from 6.24 (GmTGA14) to 9.4 (GmTGA13) (Table 1).

**Table 1:** Molecular characteristics of TGA in soybean.

Number	Gene ID number	Gene name	Amino acid residues	MW (kDa)	p*I*	Chromosome	bZIP domain	bZIP-D box
1	Glyma01G084200	*GmTGA01*	486	54.31	7.80	1	202–254	468–479
2	Glyma02G097900	*GmTGA02*	467	51.85	7.76	2	183–235	449–460
3	Glyma02G176800	*GmTGA03*	484	53.93	7.98	2	191–243	464–475
4	Glyma03G127600	*GmTGA04*	460	50.93	7.72	3	175–227	442–453
5	Glyma03G128200	*GmTGA05*	290	32.37	8.84	3	45–46	272–283
6	Glyma03G142400	*GmTGA06*	491	55.2	8.27	3	200–246	472–483
7	Glyma04G254800	*GmTGA07*	362	40.93	8.91	4	69–115	342–353
8	Glyma05G182500	*GmTGA08*	370	42.10	7.72	5	84–135	349–360
9	Glyma06G107300	*GmTGA09*	355	39.98	6.64	6	70–122	336–347
10	Glyma08G140100	*GmTGA10*	374	42.56	7.75	8	84–135	348–359
11	Glyma10G092100	*GmTGA11*	517	57.92	7.10	10	225–277	498–509
12	Glyma10G276100	*GmTGA12*	456	51.00	6.51	10	169–221	438–449
13	Glyma10G296200	*GmTGA13*	332	37.14	9.40	10	47–98	315–326
14	Glyma11G183700	*GmTGA14*	493	54.39	6.24	11	191–241	448–459
15	Glyma11G236300	*GmTGA15*	362	41.01	6.73	11	78–130	343–354
16	Glyma12G088700	*GmTGA16*	501	55.25	7.10	12	200–249	456–467
17	Glyma12G184500	*GmTGA17*	487	54.05	7.24	12	185–235	442–453
18	Glyma13G085100	*GmTGA18*	370	41.92	7.63	13	78–129	344–355
19	Glyma13G193700	*GmTGA19*	469	52.00	6.36	13	184–236	452–463
20	Glyma13G316900	*GmTGA20*	490	54.63	7.48	13	187–237	445–456
21	Glyma14G167000	*GmTGA21*	370	41.89	8.32	14	78–128	344–355
22	Glyma15G232000	*GmTGA22*	491	54.41	6.25	15	206–258	474–485
23	Glyma18G020900	*GmTGA23*	362	41.05	8.21	18	78–130	343–354
24	Glyma19G130200	*GmTGA24*	459	50.72	8.92	19	174–226	442–453
25	Glyma19G145300	*GmTGA25*	491	55.36	8.71	19	200–246	473–484
26	Glyma20G113600	*GmTGA26*	455	50.67	6.39	20	168–220	437–448
27	Glyma20G246400	*GmTGA27*	332	37.14	8.98	20	47–98	315–326


**FIGURE 1:**
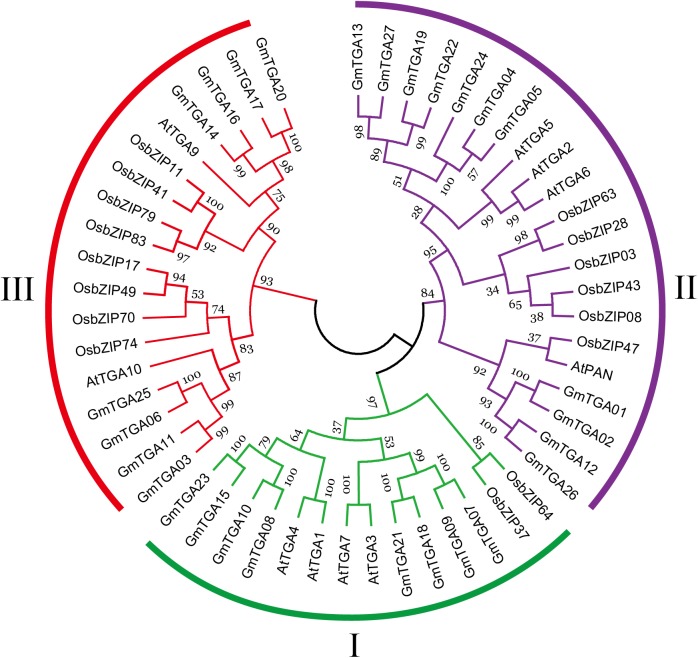
Phylogenetic analyses of TGA proteins from *Arabidopsis*, rice and soybean. Each clade is marked by a separate color.

To evaluate the evolutionary history of GmTGAs and their relationships with TGAs in other plants, the TGA protein sequences were aligned and compared with TGA sequences from *Arabidopsis* and rice, resulting in a multiple sequence alignment and phylogeny of 53 TGA proteins. The phylogenetic tree showed that 53 TGAs were divided into three major clades: Clade I, II and III (Figure 1), which agreed with previous reports on sub-classification within TGA family proteins (Murmu et al., 2010; Espín et al., 2012). Clades I and II both contain 8 GmTGAs, while clade III has 11 members of this protein family.

### Gene Structure and *Cis*-Acting Elements

To obtain some insight into the gene structure of 27 *GmTGA* genes, their intron-exon organization was examined. *GmTGA* genes were interrupted by 6–11 introns (Supplementary Figure S1). In order to better annotation and prediction of potential functions of *GmTGA* genes, we searched for *cis*-elements in their putative promoter regions. The results revealed that all *GmTGA* genes contained one or more of the ABRE, ARE, MYB, MYC, MBS, or G-box elements in their putative promoter regions. Only the putative promoter region of *GmTGA17* carried the dehydration-responsive element (DRE). Low-temperature responsive elements (LTRE) were only observed in the putative promoter regions of *GmTGA05*, *GmTGA25*, and *GmTGA27* (Supplementary Table S2).

### Domain Structure of Soybean TGA Proteins

To analyze the structural characteristics of conserved domains in GmTGA proteins, a multiple sequence alignment was performed using the 27 full-length TGA amino acid sequences from soybean. Multiple alignments showed that the GmTGAs contain a typical bZIP domain, two polyglutamine domains and a unique bZIP-D box (Supplementary Figure S2). The bZIP domain consists of a DNA binding domain and a leucine zipper domain. DNA binding domains are located in the N-terminus. Highly conserved amino acid sequences in the DNA binding domains have Glutamine, Alanine and Serine residues, which are regularly spaced by three different amino acids. The leucine zipper domain is formed by a heptad repeat of leucine residues. The GmTGAs also contain the polyglutamine domains I (QI) and II (QII). All members of GmTGAs possess the bZIP-D box, located in the C-terminus (Supplementary Figure S2B). These observations are in accordance with previous reports about the structural characteristics of the TGA conserved domains (Jakoby et al., 2002; Farinati et al., 2010; Espín et al., 2012).

### Expression Profiles of Soybean *TGA* Genes Are Different for Each Tissue

**FIGURE 2:**
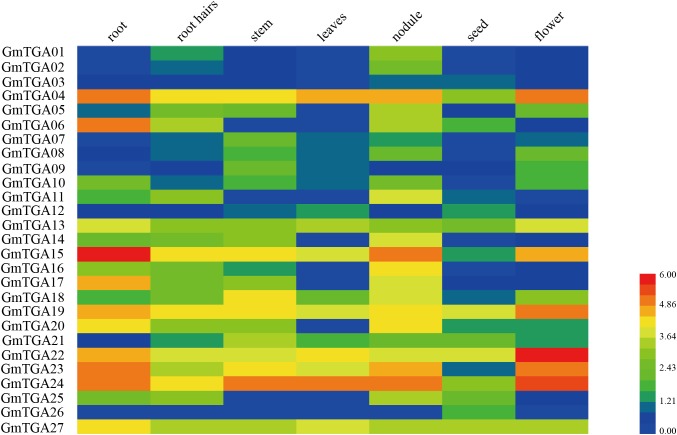
Expression profiles of soybean *TGA* genes in seven different tissues of soybean. Different colors in map represent gene transcript abundance values. The color scale is shown in the bar at bottom of figure.

To analyze the expression patterns of *GmTGA* genes, the transcriptome data of seven soybean tissues and organs was extracted from the soybean genome database and made a heatmap of *GmTGA* genes expression profile (Figure 2). A total of 26 *GmTGA* genes were expressed in all evaluated tissues, whereas *GmTGA01* were expressed in root, root hairs, stem, nodules, seed and flower tissue, but not in leaves. Additionally, the expression patterns were different between *GmTGA* genes in same tissue. For example, *GmTGA04*, *GmTGA*0*6*, *GmTGA10*, *GmTGA15*, *GmTGA17*, *GmTGA20*, *GmTGA22*, and *GmTGA23* were expressed at their highest levels in roots, the expressions of *GmTGA07*, *GmTGA*0*9*, *GmTGA18*, and *GmTGA21* were highest in stems, *GmTGA01*, *GmTGA*0*2*, *GmTGA*0*5*, *GmTGA11*, *GmTGA14*, *GmTGA16*, and *GmTGA25* were expressed most strongly in nodules, *GmTGA03*, *GmTGA12*, and *GmTGA26* transcription was most enriched in seeds, and *GmTGA08*, *GmTGA13*, *GmTGA19*, *GmTGA22*, and *GmTGA24* were found most highly expressed in flowers. The expression of *GmTGA01* reached its highest level in nodules, but was not expressed in leaves. These transcriptional patterns suggested that the expression of these genes might be governed by diverse and potentially tissue-dependent regulatory mechanisms.

### *GmTGA* Genes Are Involved in Response to Drought and Salt

**FIGURE 3:**
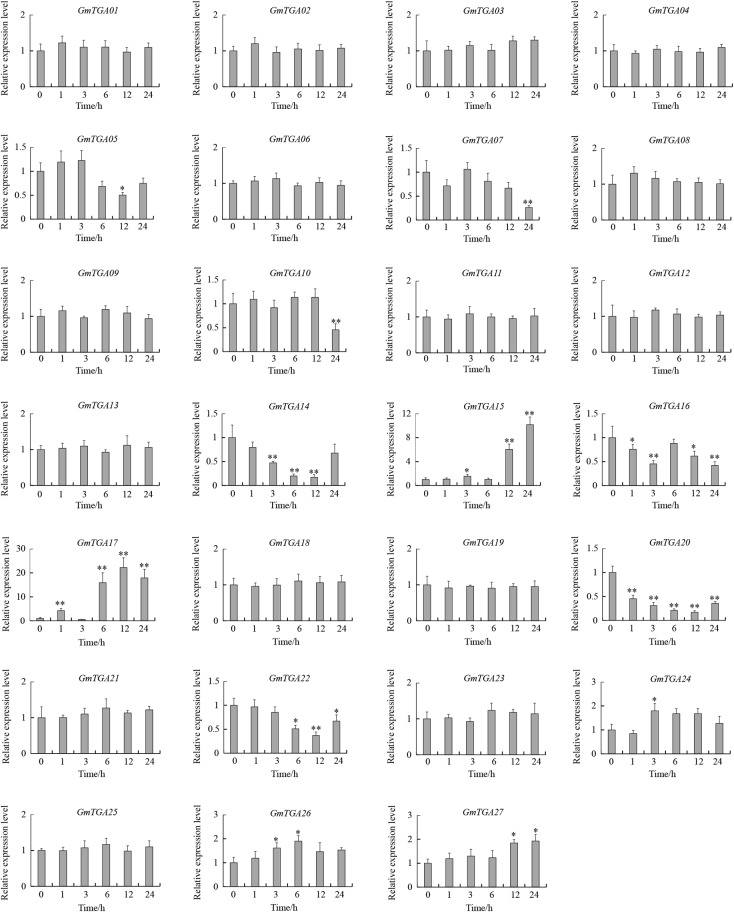
Expression profiles of soybean *TGA* genes under drought stress conditions. The expression levels were normalized to that of *CYP2.* Values are means and SD obtained from four biological replicates. The asterisks indicate a statistical significance (^∗^*P* < 0.05 and ^∗∗^*P* < 0.01) compared with the corresponding controls.

**FIGURE 4:**
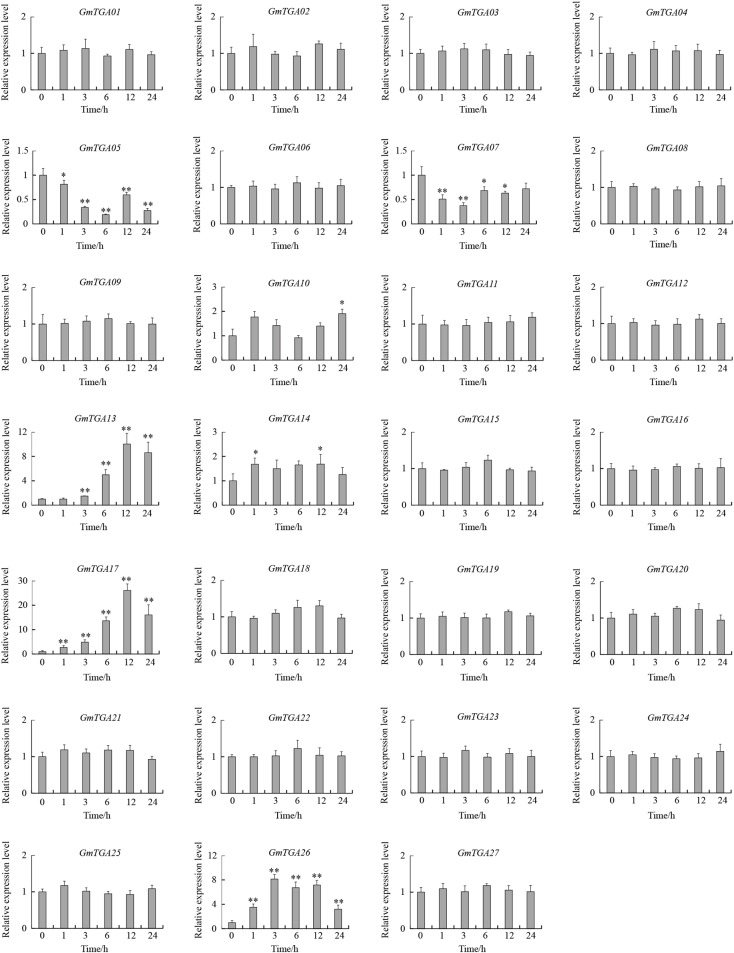
Expression profiles of soybean *TGA* genes under salt stress conditions. The expression levels were normalized to that of *CYP2.* Values are means and SD obtained from four biological replicates. The asterisks indicate a statistical significance (^∗^*P* < 0.05 and ^∗∗^*P* < 0.01) compared with the corresponding controls.

To investigate the potential roles of GmTGAs in response to multiple abiotic stresses, the expression profiles of *GmTGA* genes in soybean plants treated with drought, salt or water were examined by qRT-PCR. The data revealed that the transcript levels of *GmTGA* genes showed no obvious difference under non-stress conditions (data not show). Under drought treatment, twelve *GmTGA* genes had a significantly different transcriptional response to drought (Figure 3). Among them, *GmTGA15*, *GmTGA17*, *GmTGA24*, *GmTGA26*, and *GmTGA27* had significant increases in transcript level after drought treatment, especially *GmTGA15* (10-fold) and *GmTGA17* (22-fold), which reached a peak at 24 and 12 h post-treatment, respectively. *GmTGA05*, *GmTGA07*, *GmTGA10*, *GmTGA14*, *GmTGA16*, *GmTGA20*, and *GmTGA22* had significant decreases in transcript level after drought treatment. The other *GmTGA* genes showed no response to drought (Figure 3). Following salt treatment,; seven *GmTGA* genes had a significantly different transcriptional response to salt (Figure 4). Among them, *GmTGA10*, *GmTGA13*, *GmTGA14*, *GmTGA17*, and *GmTGA26* had significant increases in transcript level after salt treatment, especially *GmTGA13* (10-fold), *GmTGA17* (26-fold), and *GmTGA26* (8-fold), which had the highest transcript levels at 12 and 3 h post-treatment, respectively. *GmTGA05* and *GmTGA07* had significant decreases in transcript level after salt treatment. The other *GmTGA* genes had no significant changes under salt treatment (Figure 4). In light of its dramatic up-regulation in stress conditions, *GmTGA17* was selected for further study of the roles of GmTGAs in abiotic stress tolerance.

### GmTGA17 Is a Nuclear Protein With Transcriptional Activation Activity

**FIGURE 5:**
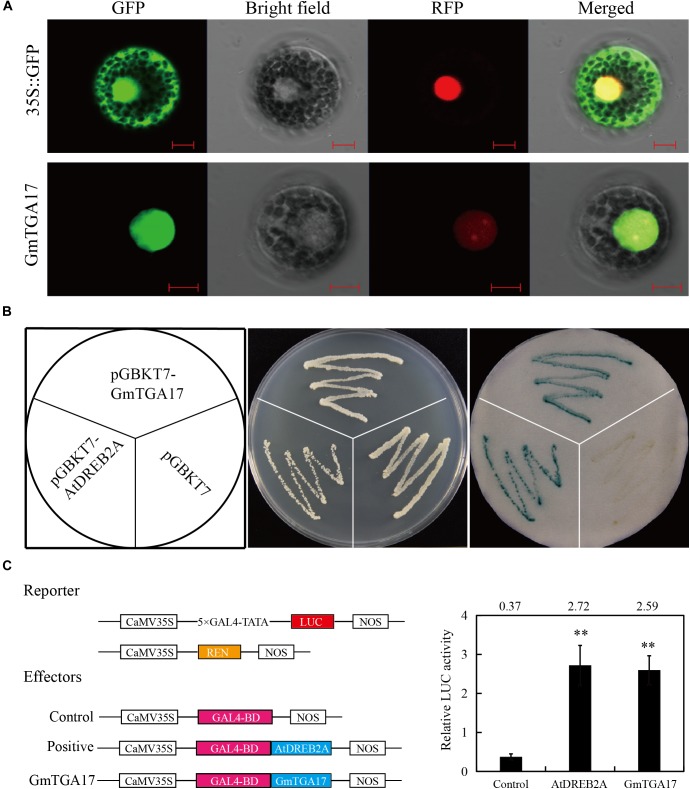
Subcellular localization and transcription activation analysis of GmTGA17. **(A)** Co-localization of GmTGA17. The recombinant plasmids of GmTGA17-GFP and NtTGA2.2-RFP were co-transformed into *Arabidopsis* protoplasts. Results were detected with confocal microscopy. Scale bars = 10 μm. **(B)** Transcriptional activity analysis of GmTGA17 in yeast cells. pGBKT7-AtDREB2A and pGBKT7 were used as positive and negative controls, respectively. **(C)** Transient expression assay in *Arabidopsis* protoplasts investigating the transcriptional activation activity of GmTGA17. The relative LUC activities in each sample was normalized relative to that of the internal control. Values are the mean ± SD of three independent replicates and the asterisks indicate a significant difference (^∗∗^*P* < 0.01) compared with the corresponding controls.

To follow the subcellular localization of GmTGA17 proteins, the ORF of *GmTGA17* was fused to GFP, as well as *NtTGA2.2* fused to RFP as a positive control, The fusion genes were subsequently co-transformed into *Arabidopsis* protoplasts. The visible fluorescence showed that GmTGA17 was located in the nucleus (Figure 5A). This finding was in accordance with its putative function as a transcription factor.

In order to test for transcriptional activity of GmTGA17, the fusion plasmids pGBKT7-GmTGA17, the positive control pGBKT7-AtDREB2A, and the negative control pGBKT7 were separately transformed into yeast strain AH109. The transformed yeast strain grew well in non-selective medium SD/–Trp and, using X-α-Gal, it was observed that both the positive control and the cells harboring pGBKT7-GmTGA17 displayed β-galactosidase activity, whereas the negative control exhibited no β-galactosidase activity (Figure 5B), suggesting that GmTGA17 possesses transcriptional activation activity in yeast cells.

To further confirm whether GmTGA17 has transcriptional activation activity, we used a dual reporter system for a transient expression assays in *Arabidopsis* protoplasts (Liu et al., 2018). In this system, the reporter plasmid was constructed by fusing the firefly luciferase (*LUC*) gene to a 5 × GAL4 binding site. The renilla luciferase (*REN*) gene under the control of the CaMV35S promoter was used as the internal control (Figure 5C). The ORF of *GmTGA17* was fused to the GAL4 binding domain (GAL4-BD) as effector plasmid (Figure 5C). After co-transformed three plasmids into *Arabidopsis* protoplasts, the relative LUC activity was determined. The results showed that the relative LUC activity was significantly upregulated when *GmTGA17* or the positive control *AtDREB2A* expressed, compared with the empty vector control (Figure 5C). The above findings suggested that GmTGA17 potentially acts as transcriptional activation in plant cell nucleus.

### Promoter Activity of *GmTGA17* During Drought and Salt Stress

**FIGURE 6:**
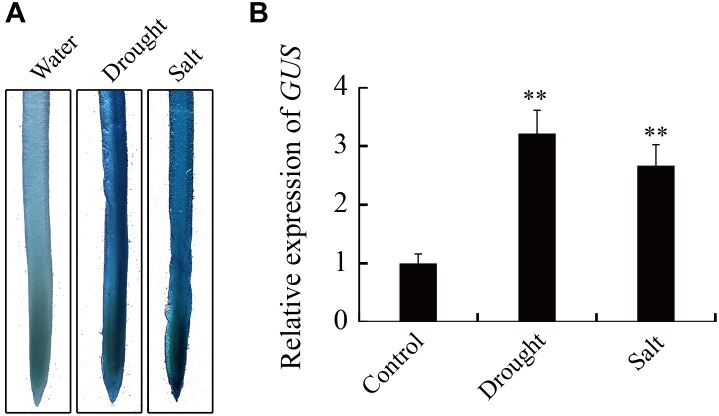
Expression of the *GUS* reporter gene under the control of *GmTGA17* promoter in transgenic soybean hairy roots. **(A)** Histochemical assay and **(B)** relative expression of *GUS.* The *GmTGA17pro*-GUS transgenic soybean hairy roots were treated with distilled water, 10% (m/v) PEG6000, or 100 mM NaCl for 6 h before being subjected to histochemical and expression analysis. The expression level of *CYP2* was used as quantitative control. Values are means and SD obtained from four biological replicates. The asterisks indicate a statistical significance (^∗∗^*P* < 0.01) compared with the corresponding controls.

Analysis by qRT-PCR showed that the expression of *GmTGA17* is responsive to drought and salt treatments. We used an established promoter-GUS construct in conjunction with a 1.9 kb *GmTGA17* promoter upstream of the transcription start site, to further study the activity of the *GmTGA17* promoter under drought and salt treatments. The construct was then transformed into soybean hairy roots. Staining of transgenic hairy roots showed that GUS activity was significantly increased in hairy roots after 6 h of drought or salt treatments compared with the hairy roots treated with water (Figure 6A). qRT-PCR date, agreed with the observations of GUS histochemical staining, showed that GUS was also up-regulated (Figure 6B). The above results suggested that the promoter activity of *GmTGA17* was enhanced by drought and salt treatments.

### Overexpression of *GmTGA17* Improved *Arabidopsis* Tolerance to Drought

**FIGURE 7:**
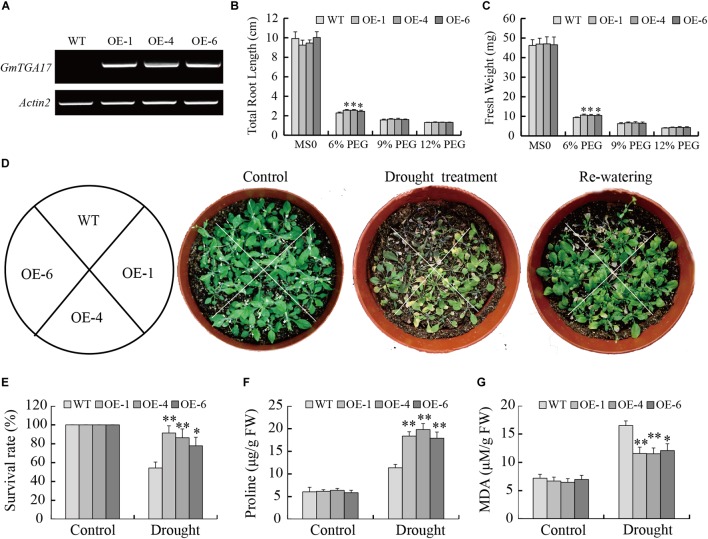
Heterologous overexpression of *GmTGA17* in *Arabidopsis* enhanced drought tolerance. **(A)** Semi-qRT-PCR analysis of *GmTGA17* expression levels in T_3_ transgenic *Arabidopsis* lines. **(B)** Total root length and **(C)** fresh weight of WT and transgenic lines exposed to 0, 6, 9, and 12% (m/v) PEG6000 for 8 days. **(D)** Phenotypes of WT and transgenic plants grown in soil under drought stress: control, treated for 2 weeks and re-watered for 1 week. **(E)** Survival rates was counted after 1 week of recovery. **(F)** Proline and **(G)** MDA contents were measured 10 days after treatment. All values are presented as means of three independent replicates (*n* = 40). The error bars indicate SD. The asterisks denote a significant difference (^∗^*P* < 0.05 and ^∗∗^*P* < 0.01) compared with the corresponding controls.

Since *GmTGA17* was identified as a predicted stress-tolerance regulator, the drought and salt tolerance phenotypes of transgenic *Arabidopsis* lines grown in medium and soil were examined. The semi-qRT-PCR result was shown in Figure 7A, and T_3_ transgenic *Arabidopsis* lines (OE-1, OE-4, and OE-6) with the high expression of *GmTGA17* were selected for further experiments (Supplementary Figure S3). PEG6000 was used to simulate drought stress when grown in medium. In the absence of PEG, the total root length and fresh weight showed no difference between transgenic and WT plants. In contrast, when exposed to 1/2 MS medium with 6% PEG6000 for 8 days, the growth of transgenic and WT plants was strongly inhibited, though the degree of inhibition in the transgenic plants was much lower than that of WT plants. The total root length and fresh weight of transgenic plants were significantly longer and greater than those of WT lines (Figure 7B,C). The total root length and fresh weight were not significantly different between transgenic lines and WT plants when grown on 1/2 MS medium supplemented with 9% and 12% PEG (Figure 7B,C).

To compare with the above results, we also tested the tolerance of transgenic *Arabidopsis* lines growing in the soil under drought stress conditions. After a 2-week water-deficit regimen, the leaves of WT plants became shriveled or died, while transgenic lines were only slightly shriveled and showed lower mortality than WT plants (Figure 7D). Compared to a 54.32% survival rate of WT plants, the survival rate of transgenic plants was 77.78–91.35% after 1 week of re-watering following treatment (Figure 7E). After a 10-day water-withholding treatment, two stress-related parameters, proline and MDA contents, were compared between the transgenic and WT seedlings. The results showed that proline and MDA contents in transgenic seedlings (17.90–18.37 μg/g FW and 11.52–12.07 μM/g FW) were significantly higher and lower than in WT plants (11.37 μg/g FW and 16.49 μM/g FW) under drought stress conditions, respectively (Figure 7F,G). Collectively, these results indicated that *GmTGA17* may have a potential function in enhancement of the transgenic plants tolerance to drought stress.

### Overexpression of *GmTGA17* Improved *Arabidopsis* Tolerance to Salinity

**FIGURE 8:**
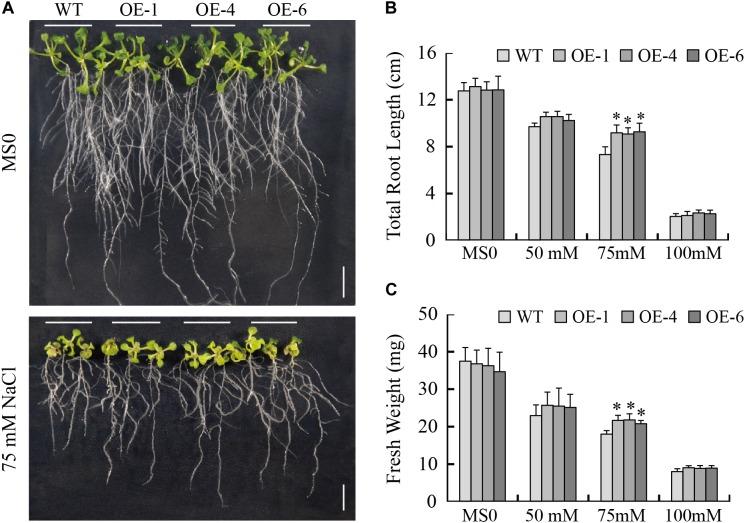
Response of WT and transgenic *Arabidopsis* plants to NaCl treatment. **(A)** Phenotypes of WT and transgenic seedlings under NaCl treatment. The photographs were taken after 8 days of treatment. **(B)** Total root length and **(C)** fresh weight of WT and transgenic lines exposed to 0, 50, 75, and 100 mM NaCl for 8 days. Scale bars = 1 cm. All values are presented as means of three independent replicates (*n* = 30). The error bars indicate SD. The asterisks denote a significant difference (^∗^*P* < 0.05) compared with the corresponding controls.

To determine if *GmTGA17* is in involved in responses to salt stress, we tested the salt tolerance of the transgenic *Arabidopsis* lines. When grown on 1/2 MS medium, the no difference in phenotype was observed between the transgenic and WT seedlings (Figure 8A). When grown on 1/2 MS medium containing 75 mM NaCl for 1 week, the total root length of transgenic plants was significantly longer than those of WT plants (Figure 8B). Similarly, the fresh weight of transgenic plants was significantly greater than those of WT plants (Figure 8C). In response to 50 or 100 mM NaCl treatments, the total root length and fresh weight were different between the transgenic and WT seedlings, though not significant at the 0.05 level (Figure 8B,C).

**FIGURE 9:**
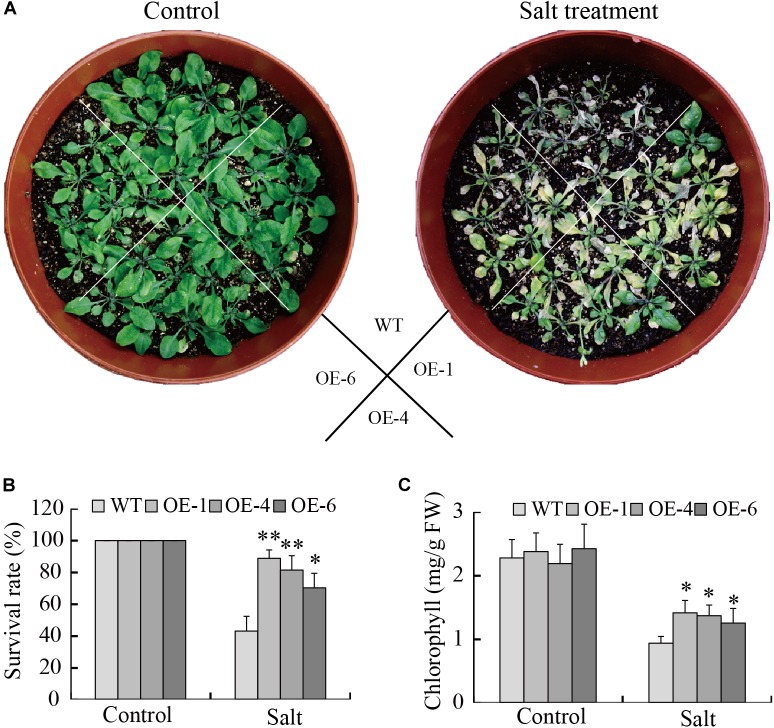
Enhance salt tolerance mediated by heterologous overexpression of *GmTGA17* in *Arabidopsis*. **(A)** Phenotypes of WT and transgenic plants grown in soil under high salinity treatment conditions. The photographs were taken after 1 week of treatment. **(B)** Survival rates was counted after 1 week of treatment. **(C)** Chlorophyll content was measured 5 days after treatment. All values are presented as means of three independent replicates (*n* = 40). The error bars indicate SD. The asterisks denote a significant difference (^∗^*P* < 0.05 and ^∗∗^*P* < 0.01) compared with the corresponding controls.

The capacity of *GmTGA17* transgenic lines to respond to high salinity stress was also assessed. The leaves of WT plants were severely wilted, whereas, the leaves of transgenic plants were slightly damaged but still remained green after treated for 1 week (Figure 9A). The transgenic plants displayed a significantly higher survival rate compared with WT plants under salinity conditions (Figure 9B). We also analyzed the chlorophyll content of all experimental plants and observed a significantly higher chlorophyll content in the transgenic plants compared with WT plants (Figure 9C). Together, these results demonstrated that overexpression of *GmTGA17* enhanced tolerance to salt stress in transgenic *Arabidopsis* plants.

### *GmTGA17* Promoted ABA-Induced Stomatal Closure

**FIGURE 10:**
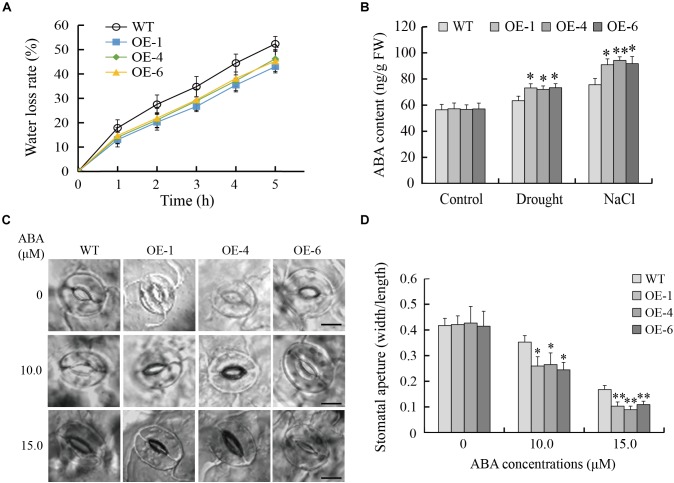
*GmTGA17* promotes ABA-induced stomatal closure. **(A)** Water loss from detached leaves of transgenic *Arabidopsis* lines and WT. The water loss of 1.0 g detached leaves from different lines was expressed as the percentage of initial fresh weight. Values are presented as means of three independent replicates. The error bars indicate SD. **(B)** Endogenous ABA content in leaves of transgenic *Arabidopsis* lines and WT plants before and after different abiotic stress treatments. ELISA was used to measure ABA content in leaves of transgenic *Arabidopsis* lines and WT plants with drought treatment for 10 days or salt treatment for 5 days. Values are presented as means ± SD of three independent replicates. The asterisks denote a significant difference (^∗^*P* < 0.05 and ^∗∗^*P* < 0.01) compared with the corresponding controls. **(C,D)** ABA-mediated stomatal closure. Rosette leaves from 3-week-old transgenic *Arabidopsis* lines and WT plants were treated with stomatal opening solution and in ABA solution (0, 10 or 15 μM) for another 3 h. Scale bars = 10 μm. Data are means of three independent replicates (*n* = 40). The error bars indicate SD. The asterisks denote a significant difference (^∗^*P* < 0.05 and ^∗∗^*P* < 0.01) compared with the corresponding controls.

A reduced water loss rate is a major factor contributing to the maintenance of moisture under drought stress. In the present study, data showed that the detached leaves from transgenic *Arabidopsis* lines lost water more slowly than those of WT plants (Figure 10A). Since the stomatal aperture has a close link with water loss, we examined the stomata of transgenic *Arabidopsis* and WT plants with or without ABA treatment. Under normal growth conditions, there was no obvious difference in stomatal aperture between transgenic *Arabidopsis* and WT plants. In the presence of 10 or 15 μM ABA, the stomatal aperture of transgenic *Arabidopsis* plants was significantly smaller than that of WT plants (Figure 10C,D). Therefore, transgenic *Arabidopsis* lines showed more rapid ABA-induced stomatal closure than did WT plants.

In addition, endogenous ABA content were measured in transgenic *Arabidopsis* lines and WT plants before and after stress treatments. As shown in Figure 10B, there was no significant difference in ABA content in leaves between transgenic *Arabidopsis* and WT plants under normal growth conditions. However, in response to drought or salt stress, the leaves of transgenic *Arabidopsis* lines accumulated significantly higher content of ABA than WT plants, suggesting that drought- and salt-tolerance phenotypes of transgenic *Arabidopsis* lines are at least partially derived from higher endogenous ABA content.

### *GmTGA17* Upregulated ABA-Responsive Genes in Transgenic *Arabidopsis*

**FIGURE 11:**
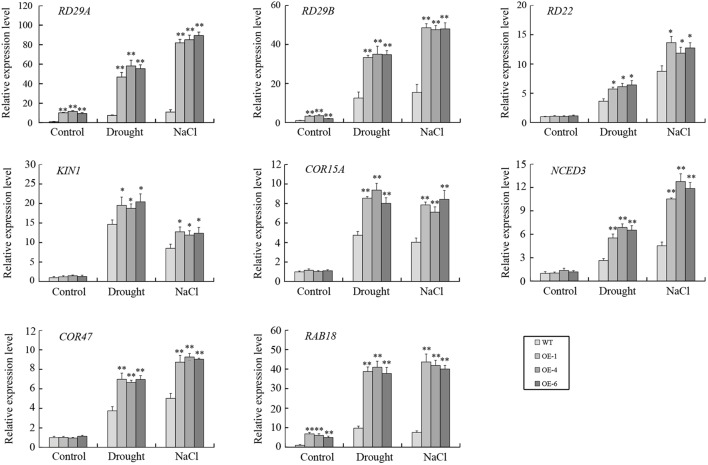
The expressions of several ABA-responsive genes in transgenic *Arabidopsis* lines and WT plants following drought and salt stress. qRT-PCR was used to detect expression levels of stress-related genes in transgenic *Arabidopsis* lines and WT plants with drought treatment for 10 days or salt treatment for 5 days. Values are means and SD obtained from four biological replicates. The asterisks indicate a statistical significance (^∗^*P* < 0.05 and ^∗∗^*P* < 0.01) compared with the corresponding controls.

To further elucidate the possible mechanism of *GmTGA17* during the abiotic stress response, the transcript levels of eight known ABA-responsive genes, i.e., *RD29A*, *RD29B*, *RD22*, *KIN1*, *COR15A*, *NCED3*, *COR47*, and *RAB18*, were analyzed in transgenic *Arabidopsis* lines and WT plants under drought or salt stress treatment. The qRT-PCR data showed that the expressions of *RD29A*, *RD29B*, and *RAB18* were significantly up-regulated in transgenic *Arabidopsis* lines compared with WT plants under both non-stress conditions and stress conditions (Figure 11). Under non-stress conditions, no obvious differences in the expressions of *RD22*, *KIN1*, *COR15A*, *NCED3*, and *COR47* were observed between transgenic *Arabidopsis* lines and WT plants. However, the transcript levels of *RD22*, *KIN1*, *COR15A*, *NCED3*, and *COR47* were significant higher in transgenic *Arabidopsis* lines than those in WT plants under both drought and salt treatments (Figure 11).

### *GmTGA17* Improves Drought and Salt Stress Tolerance in Transgenic Soybean Hairy Roots

**FIGURE 12:**
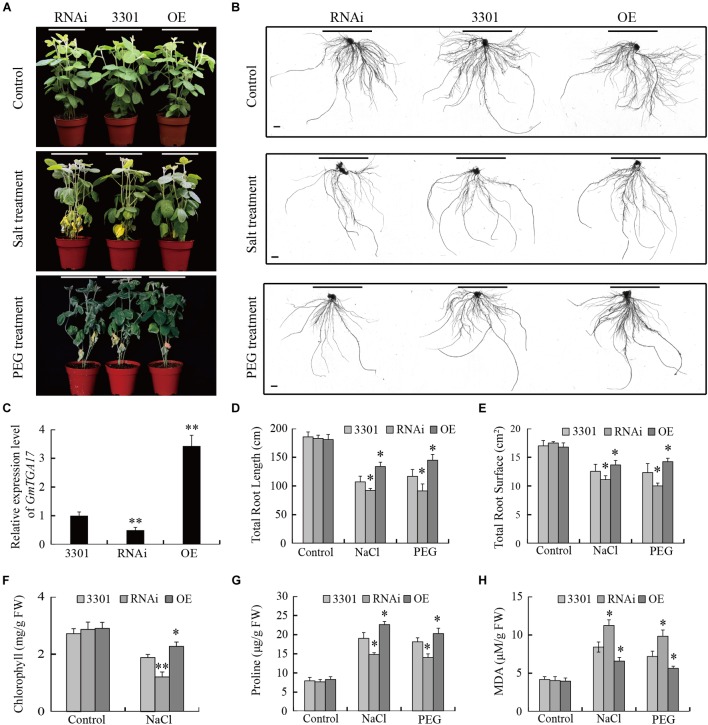
Performance of *GmTGA17* transgenic soybean hairy roots under drought and salt stress. **(A)** Phenotypes of plants with transgenic soybean hairy roots under PEG and high salinity treatments. **(B)** Transgenic hairy root images: control and after treatment. Scale bars = 1 cm. **(C)** Relative *GmTGA17* expression in roots overexpressing *GmTGA17*, RNA interference hairy roots, and control roots as shown by qRT-PCR. qRT-PCR quantifications were normalized to the expression of *CYP2*. Values are means and SD obtained from four biological replicates. The asterisks indicate a statistical significance (^∗∗^*P* < 0.01) compared with the corresponding controls. **(D,E)** Total root length and total root surface were measured 10 days after treatments **(F–H)** Chlorophyll, proline, and MDA contents in leaves of plants with transgenic hairy roots were measured 1 week after treatments. All values are presented as means of three independent replicates (*n* > 45). The error bars indicate SD. The asterisks denote a significant difference (^∗^*P* < 0.05 and ^∗∗^*P* < 0.01) compared with the corresponding controls.

The participation of *GmTGA17* in drought and salt tolerance was further investigated by performing similar abiotic stress assays in *Agrobacterium rhizogenes*-mediated soybean hairy roots (Collier et al., 2005; Kereszt et al., 2007; Sun et al., 2015; Wang et al., 2015). qRT-PCR analysis showed that the expression level of *GmTGA17* was significantly higher in hairy roots overexpressing *GmTGA17*, and significantly lower in RNAi hairy roots suppressing *GmTGA17* compared with the empty vector control (Figure 12C).

Subsequently, we evaluated the drought and salt tolerance in plants with transgenic hairy root grown in soil watered with 20% (m/v) PEG or 150 mM NaCl solutions for 10 days. To avoid damaging the transgenic hairy roots, PEG6000 was also used for simulating drought stress instead of withholding water in this assay. All plants grew well and displayed a similar phenotype under non-stress conditions (Figure 12A). Under high-salinity, the leaves of all experimental plants gradually yellowed and shriveled, while under PEG treatments, the leaves of all experimental plants wilted and bleached from bottom to top. Moreover, the plants carrying the *GmTGA17* RNAi hairy roots were the most sensitive to high salinity and PEG stress, while less susceptible were the empty vector control hairy roots, and least susceptible were the *GmTGA17* overexpression hairy roots (Figure 12A,B). Under high-salinity stress, we separately observed a significantly higher and lower chlorophyll content in leaves of plants with overexpression hairy roots and RNAi hairy roots compared with that of the empty vector control (Figure 12F). Under both stress conditions, proline content was higher in soybean plants with overexpression hairy roots but lower in RNAi lines relative to the empty vector control. Similarly, MDA content in plants with overexpression hairy roots were lower but higher in RNAi lines relative to the empty vector control (Figure 12G,H). Additionally, compared with the empty vector control, the total root length and root surface of the overexpression hairy roots were longer and greater, but the total root length of the RNAi hairy roots were shorter with decreased surface area under high-salinity and PEG stress conditions (Figure 12D,E). These results indicated that *GmTGA17* positively regulated tolerance to drought and salt stress in transgenic soybean hairy roots.

## Discussion

TGA transcription factors have been reported to function in various biological processes in plants. However, there is no comprehensive analysis of TGA transcription factors in soybean, and especially concerning their participation in abiotic stress tolerance. In the present study, a total of 27 TGA members were identified in the soybean genome. The GmTGAs could be clustered together with TGAs from *Arabidopsis* and rice in the same clade (Figure 1), suggesting that the evolution of *TGA* genes are conserved between monocots and dicots. However, the number of TGAs in soybean is expanded compared to that in *Arabidopsis*, rice, maize, and papaya (Jakoby et al., 2002; Nijhawan et al., 2008; Espín et al., 2012; Wei et al., 2012). Gene structure analysis showed that putative *GmTGA* genes within the same clade share a similar intron-exon organization (Supplementary Figure S1), and similar patterns were observed in *Arabidopsis* and maize (Murmu et al., 2010; Wei et al., 2012). This indicates that the diverse status of intron and exon splicing might thus be meaningful for the evolution of *TGA* genes. All predicted GmTGA proteins possessed QI and QII domains, which is reported to function as a transcriptional activation domain (Schindler et al., 1992; Schwechheimer et al., 1998; Chuang et al., 1999). Additionally, we found that GmTGA17 showed transcriptional activation activity (Figure 5A,C). Taken together, we speculate that GmTGAs may act as transcription activator. TGA proteins have a conserved bZIP-D box in addition to the common domains of bZIP proteins (Jakoby et al., 2002; Farinati et al., 2010). Although the bZIP-D box is a characteristic domain for TGA family, there are currently no articles of which we are aware reporting the association of this feature with any particular biological function.

It is well known that *cis*-elements in the promoter region of a given gene are closely related to the biological functions of gene product (Cheng et al., 2013). In the present study, the *cis*-acting elements analysis revealed that the putative promoter regions of all *GmTGA* genes possess ERE, W-box, P-box or WUN-motif pathogen-related *cis*-elements (Raventós et al., 1995; Choi et al., 2004), suggesting involvement of *GmTGA* genes in defense against pathogens. This observation was in accordance with the fact that *TGA* genes involved in response to pathogen attack (Jakoby et al., 2002; Wei et al., 2012). Moreover, a number of stress-related *cis*-elements have been identified in the putative promoter regions of *GmTGA* genes (Supplementary Table S2), with evidence demonstrating participation of these *cis*-elements in responses to drought, salt, ABA, and low temperature (Baker et al., 1994; Yamaguchi-Shinozaki and Shinozaki, 1994; Busk and Pagès, 1998; Gómez-Cadenas et al., 2001; Maestrini et al., 2009). The expression profiles of these genes in different plant tissues showed that 26 deduced *GmTGA* genes were expressed in roots, root hairs, stems, leaves, nodules, seeds, and flowers, with the exception of *GmTGA01*, which had no expression in leaves (Figure 2). It is thus evident that *GmTGA* genes likely play a broad role in soybean development.

We hypothesized that the existence of abundant stress-related *cis*-elements suggests that *GmTGA* genes may participate in plant responses to a range of abiotic stresses. Our qRT-PCR data confirmed this hypothesis, with results showing that *GmTGA* genes are involved in drought and salt stress responses, though the response mechanisms are diverse. For example, *GmTGA15*, *GmTGA17* and *GmTGA25* were up-regulated, while *GmTGA05* and *GmTGA07* were down-regulated by drought and salt treatments. *GmTGA10* and *GmTGA14* were up-regulated by drought treatment, but down-regulated by salt treatment. Similar stress response mechanisms were observed in bZIP family genes of rice and *Brachypodium distachyon* (Nijhawan et al., 2008; Liu and Chu, 2015).

Using transgenic *Arabidopsis* and soybean hairy root assays, we further characterized the roles of *GmTGA17* in abiotic stress tolerance. Our data showed that *GmTGA17* could confer tolerance to drought and salinity in transgenic plants (Figure 7–9, 12). Plant tolerance to abiotic stresses are modulated by complicated plant hormone signal transduction pathways and metabolism, especially the main stress hormone ABA (Huang et al., 2008). Drought and salt stress can induce the accumulation of ABA, and this in turn regulates the expression of stress-responsive genes to improve the plant adaption to stress (Xiong et al., 2002). In this study, endogenous ABA content was significantly higher in transgenic *Arabidopsis* seedlings than WT plants under drought and salt stress treatments (Figure 10B). Coincidentally, the transcript level of a key ABA-biosynthesis gene, *AtNCED3*, was significantly up-regulated in drought- and salt-treated transgenic *Arabidopsis* lines compared with WT plants (Figure 11). Based on our stomatal aperture assay data, we speculated that transgenic *Arabidopsis* plants exhibited reduced leaves water loss due to induced ABA accumulation. Additionally, qRT-PCR data revealed that *GmTGA17* also had an induced expression response to ABA treatment (Supplementary Figure S4), and upregulated several ABA-responsive genes in transgenic *Arabidopsis* lines under drought and salt stress treatments (Figure 11). The above results suggest that *GmTGA17* may participate in plant response to drought and salinity in an ABA-dependent pathway.

Previously, AtTGA1-AtTGA7 were grouped within three clades associated with plant defense, based on sequence homology (Xiang et al., 1997; Kesarwani et al., 2007). However, the other three AtTGA members were not included in this classification. Subsequently, TGAs from *Arabidopsis* and papaya were divided into three main clades (I, II and III) according to the similarity of their protein sequences (Murmu et al., 2010; Espín et al., 2012). Clade I contains AtTGA1, AtTGA3, AtTGA4, AtTGA7, CpTGA1, and CpTGA3. AtTGA2, AtTGA5, AtTGA6, AtPAN, CpTGA2, and CpTGA4 belong to clade II. AtTGA9, AtTGA10 clustered together with CpTGA5 and CpTGA6 in the clade III.

Our phylogenetic analysis revealed that the 27 predicted GmTGAs could also be divided into clades I, II, and III, mentioned above (Figure 1). In clade I, *AtTGA3* and *AtTGA4* participate in plant pathogen response and root development, as well as playing a vital role in the regulation of response various abiotic stresses (Kesarwani et al., 2007; Alvarez et al., 2014; Du et al., 2014; Zhong et al., 2015; Canales et al., 2017). *CpTGA3* was shown to be responsive to Salicylic Acid, suggesting its potential involvement in the defense response (Espín et al., 2012). qRT-PCR data generated in this study showed that *GmTGA07*, *GmTGA10*, and *GmTGA15* are involved in responses to drought or salt stress.

In clade II, *AtPAN* is a key regulator in the control of floral patterning (Running and Meyerowitz, 1996; Chuang et al., 1999). *AtTGA2*, *AtTGA5* and *AtTGA6* play important roles in disease resistance and development (Zhang et al., 2003; Mueller et al., 2008; Stotz et al., 2013). Our results revealed that the expression of *GmTGA05*, *GmTGA13*, *GmTGA22*, *GmTGA24*, *GmTGA26*, and *GmTGA27* were up- or down-regulated by drought and salt stress. Interestingly, *GmTGA13*, *GmTGA22*, and *GmTGA24* were expressed most strongly in flowers (Figure 2), this indicates that they might be also involved in floral development processes in soybean.

Of the TGAs assigned to clade III, *AtTGA9* and *AtTGA10* were shown to participate in anther development as well as ROS-mediated responses to pathogens (Murmu et al., 2010; Noshi et al., 2016). *CpTGA5* was strongly expressed, but only in petals, suggesting an associated role in floral development (Espín et al., 2012). *OsbZIP41* was found to be up-regulated in seedlings under blue light treatment while *OsbZIP79* and *OsbZIP83* were down-regulated under abiotic stress conditions (Nijhawan et al., 2008). Miyamoto et al. (2015) found that *OsbZIP79* suppressed production of the diterpenoid phytoalexin, an antimicrobial metabolite. Among the *GmTGA* genes, the expression of *GmTGA14*, *GmTGA16*, *GmTGA17*, and *GmTGA20* were activated or repressed under drought and salt stress. Moreover, *GmTGA17* is a positive regulator of plant tolerance to drought and salt stress. The above findings suggested the functional diversification of a certain *TGA* gene or the TGA members in the same clade. However, further studies are needed to determine the specific functions of the GmTGA family genes by additional experiments.

## Conclusion

Twenty-seven soybean *TGA* genes were identified in the soybean genome. Expression analysis showed that soybean TGA family genes may participate in responses to drought and salt stress. *GmTGA17* conferred tolerance to drought and salt stress in both transgenic *Arabidopsis* plants and soybean hairy roots. However, RNAi hairy roots silenced for *GmTGA17* exhibited an increased sensitivity to drought and salt stress. Taken together, soybean *TGA* genes may act as important components of abiotic stress tolerance in plants.

## Author Contributions

Z-SX coordinated the project, conceived and designed the experiments, and edited the manuscript. BL performed the experiments and wrote the first draft. YL and J-DF conducted the bioinformatic work and performed the experiments. W-JZ, J-HL, L-GJ, Y-BZ, MC and D-HM contributed with valuable discussions. X-YC revised and edited the manuscript. Y-ZM coordinated the team. All authors read and approved the final manuscript.

## Conflict of Interest Statement

The authors declare that the research was conducted in the absence of any commercial or financial relationships that could be construed as a potential conflict of interest.

## Supplementary Material

Figure S1 Phylogenetic analysis and intron/exon structures of *TGA* genes in soybean. The phylogenetic tree (left panel) was created using MEGA6.0 software. Exon/intron structures of *TGA* genes are shown in the right panel. Gene models were drawn to scale as indicated on the bottom.

Figure S2 Structure and alignment of GmTGA proteins. **(A)** Primary structure of TGA (Jakoby et al., 2002). **(B)** Alignment of GmTGA amino acid sequences. Black arrows indicate Glutamine, Alanine, and Serine residues. Asterisks indicate the leucine zipper domain.

Figure S3 PCR detection and the expression levels of *GmTGA17* in transgenic *Arabidopsis* lines overexpressing *GmTGA17.* **(A)** Genomic DNA PCR detection of *GmTGA17* in transgenic *Arabidopsis* lines (OE-1, OE-4, and OE-6). “+” represents positive control; “–” represents negative control (WT plant). The size of target band is 671 bp. **(B)** Relative expression levels of *GmTGA17* in transgenic *Arabidopsis* lines. *Actin2* was used as a control.

Figure S4 Expression profile of *GmTGA17* under ABA treatment. Expression pattern of *GmTGA17* in soybean seedlings under ABA treatment. The expression levels were normalized to that of *CYP2.* All values are means and SD obtained from four biological replicates. The asterisks indicate a statistical significance (^∗∗^*P* < 0.01) compared with the corresponding controls.

Table S1 Primers and sequences used in this study.

Table S2 *Cis*-elements in promoters of *GmTGA* genes.

## Abbreviations

ABA: abscisic acid
ABRE: ABA-responsive element
ARE: anaerobic responsive element
ERE: elicitor-response element
FW: fresh weight
GFP: green fluorescent protein
MS: Murashige and Skoog
ORF: open reading frame
P-box: pathogen-inducible box
qRT-PCR: quantitative real-time-PCR
W-box: wound-inducible box
WT: wild type
