# Supplementary figures and images for: Genome-Wide Characterization and Expression Analysis of Soybean TGA Transcription Factors Identified a Novel TGA Gene Involved in Drought and Salt Tolerance
Source: Front Plant Sci. 2019 May 16;10:549. doi: 10.3389/fpls.2019.00549 (PMC6531876; doi:10.3389/fpls.2019.00549)

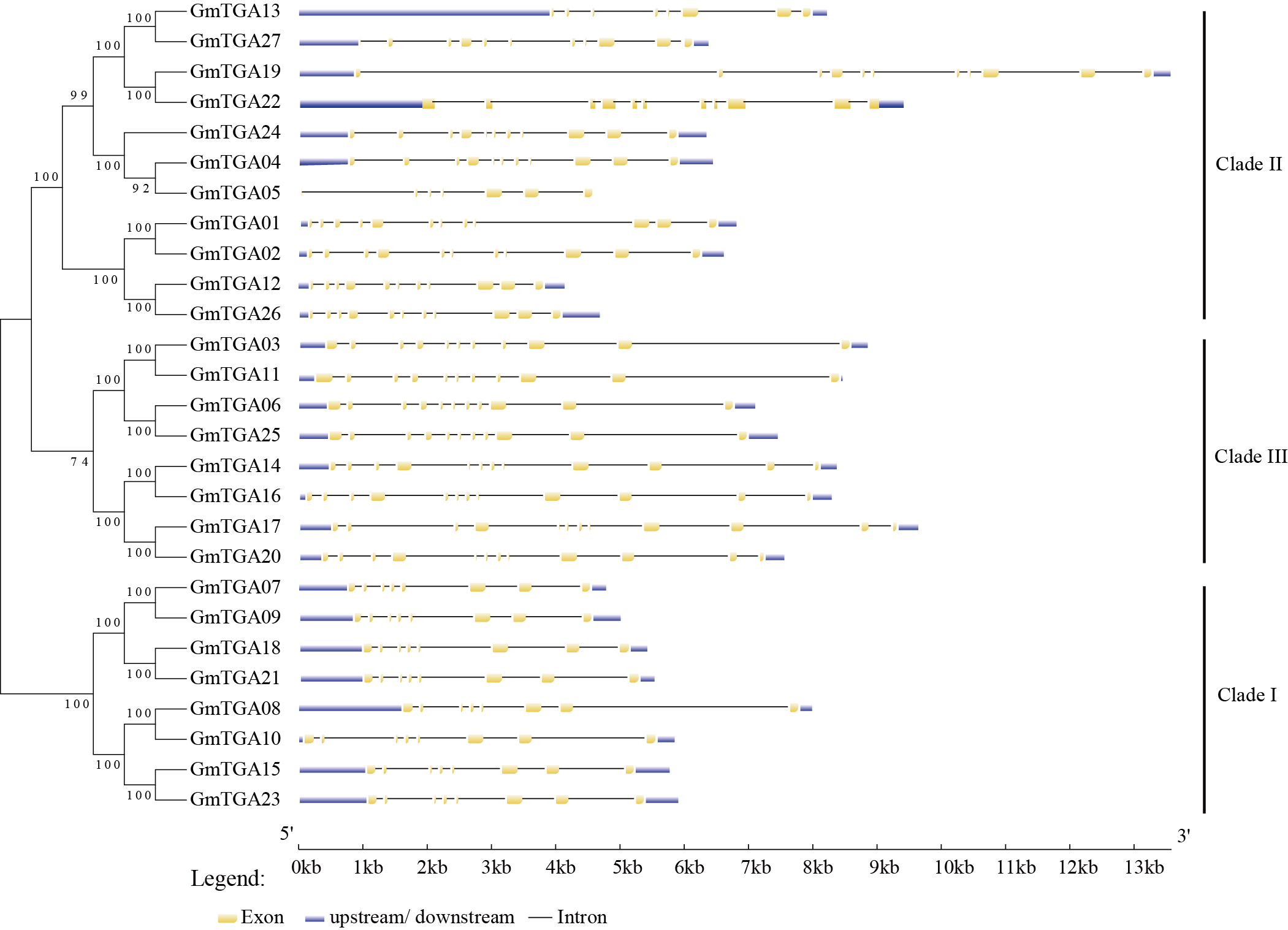

Supplement: Figure S1 — Phylogenetic analysis and intron/exon structures of TGA genes in soybean. The phylogenetic tree (left panel) was created using MEGA6.0 software. Exon/intron structures of TGA genes are shown in the right panel. Gene models were drawn to scale as indicated on the bottom. [file Image_1.TIF]

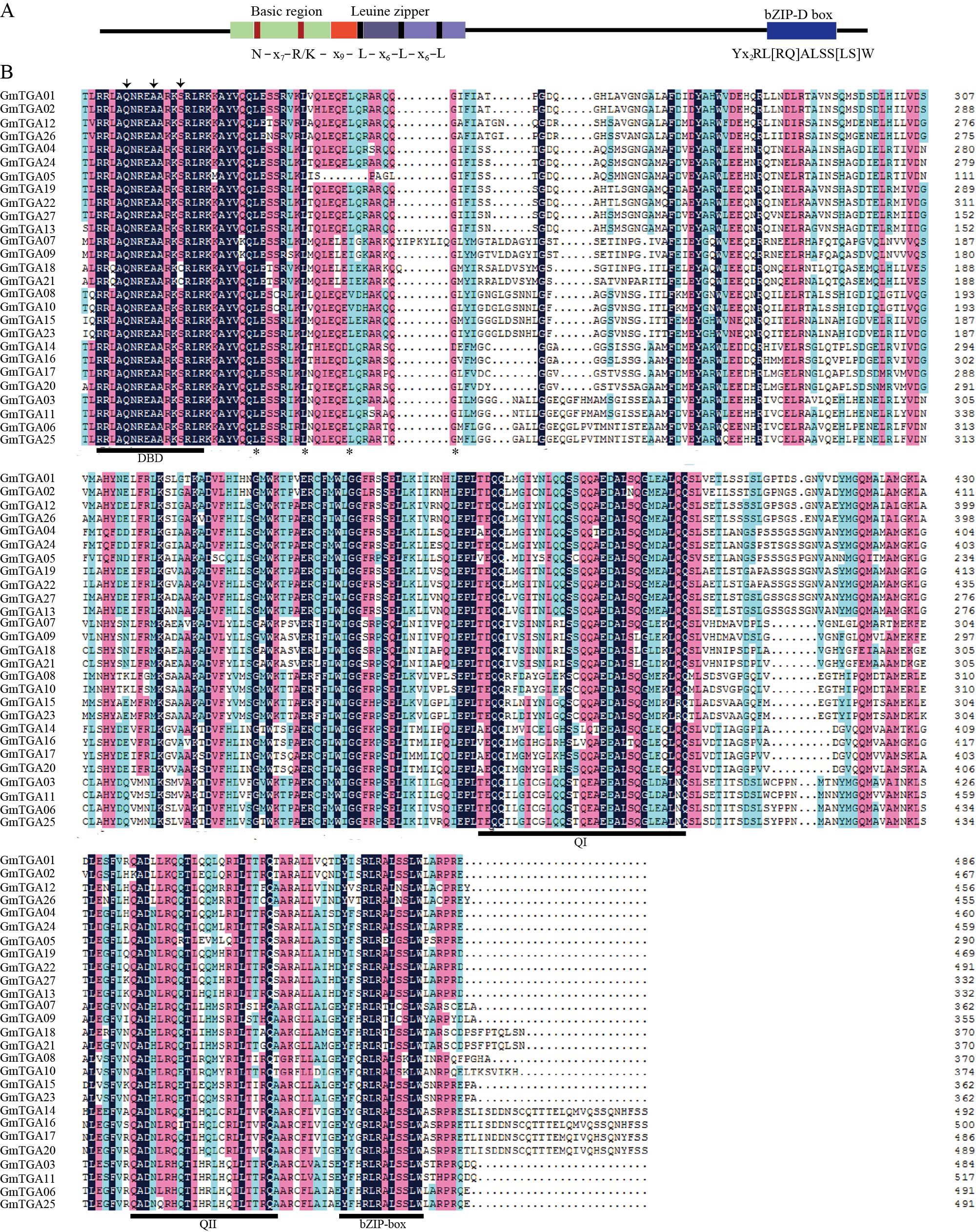

Supplement: Figure S2 — Structure and alignment of GmTGA proteins. (A) Primary structure of TGA (Jakoby et al., 2002). (B) Alignment of GmTGA amino acid sequences. Black arrows indicate Glutamine, Alanine, and Serine residues. Asterisks indicate the leucine zipper domain. [file Image_2.TIF]

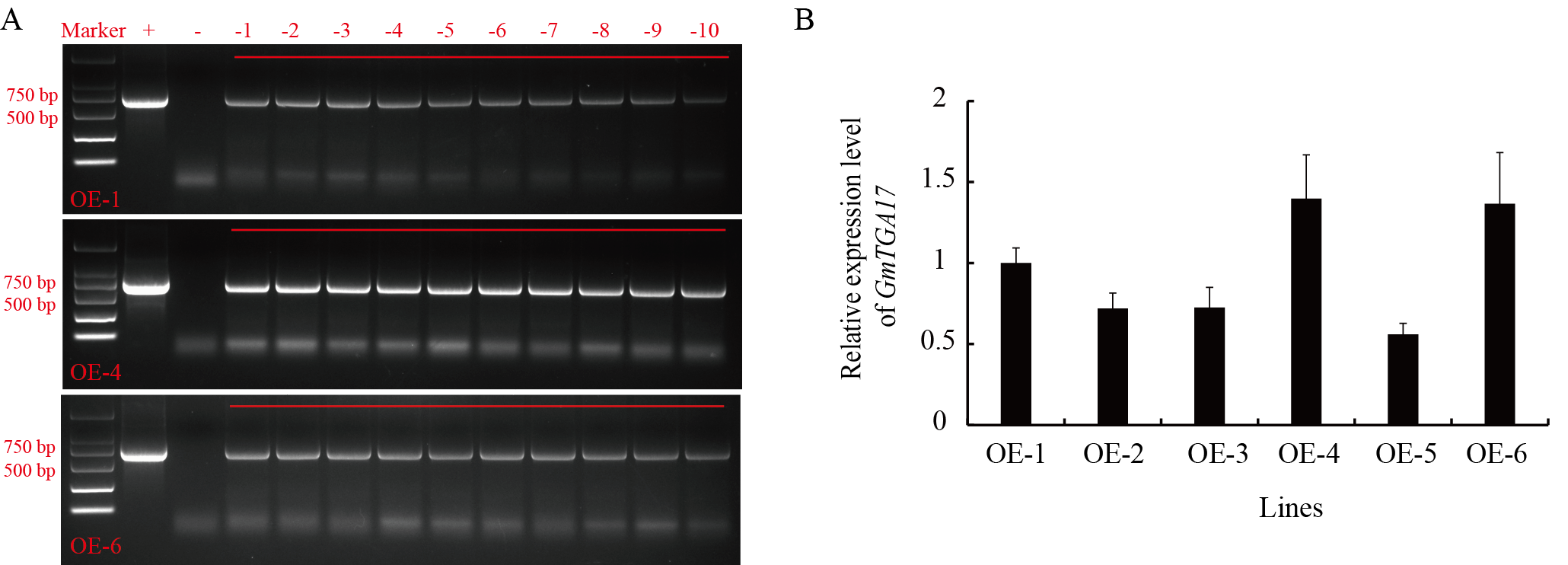

Supplement: Figure S3 — PCR detection and the expression levels of GmTGA17 in transgenic Arabidopsis lines overexpressing GmTGA17. (A) Genomic DNA PCR detection of GmTGA17 in transgenic Arabidopsis lines (OE-1, OE-4, and OE-6). “+” represents positive control; “–” represents negative control (WT plant). The size of target band is 671 bp. (B) Relative expression levels of GmTGA17 in transgenic Arabidopsis lines. Actin2 was used as a control. [file Image_3.TIF]

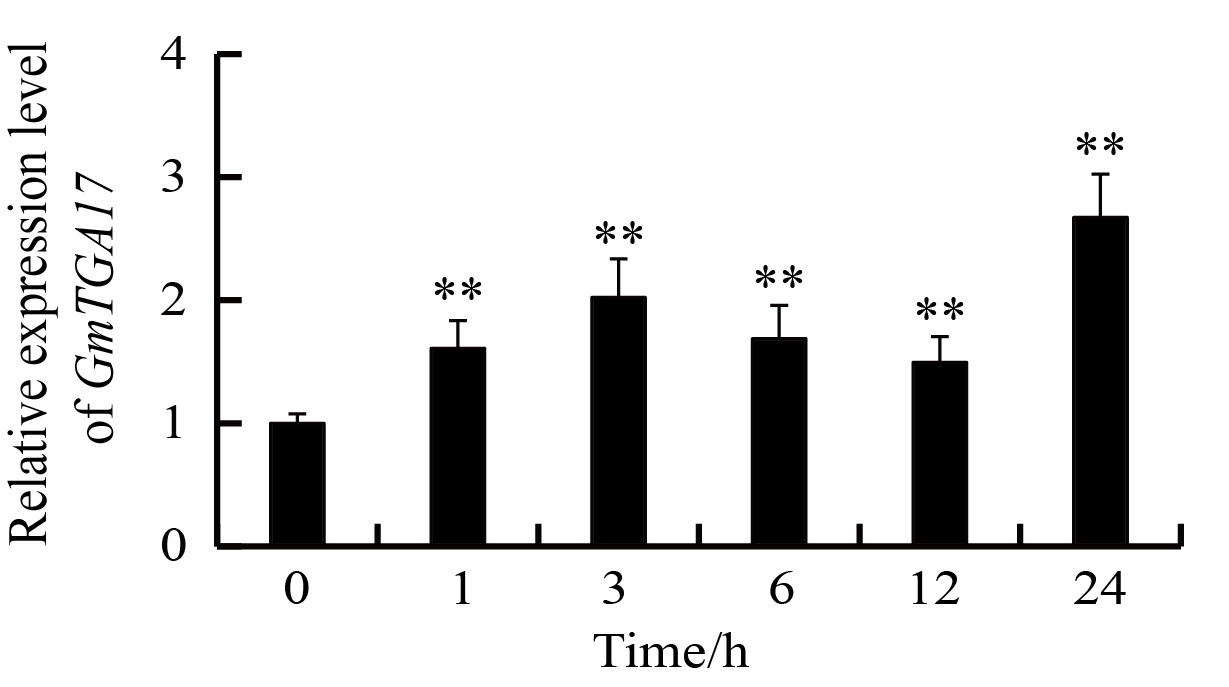

Supplement: Figure S4 — Expression profile of GmTGA17 under ABA treatment. Expression pattern of GmTGA17 in soybean seedlings under ABA treatment. The expression levels were normalized to that of CYP2. All values are means and SD obtained from four biological replicates. The asterisks indicate a statistical significance (∗∗P < 0.01) compared with the corresponding controls. [file Image_4.TIF]
